# A Shocking Occurrence

**Published:** 1884-09

**Authors:** 


### A SHOCKING OCCURRENCE.

A most painful accident (if the results of criminal carelessness may be called accidental), occurred during the late meeting of the American Dental Association. Dr. W. R. Clifton, of Waco, Texas, an honored member of the society, was in attendance, and had brought from his distant home his only and motherless child, a bright little boy of about five years of age. The father left his little prattler at the hotel, and in one short hour its poor, broken, mangled, almost lifeless body, was placed in his arms. Accompanied by its nurse, the child was crossing the street, when a carriage, driven at a reckless speed, crashed upon them. The nurse barely escaped, but the little one was trodden beneath the feet of the horses. Surgeons were summoned, when it was found that the temporal bone was crushed, the lower maxillary and the clavicle broken, besides other injuries. The skull was trepanned, and numerous portions of the bone removed, when the child recovered consciousness. Announcement of the accident was made in the Association, and a vote of sympathy with Dr. Clifton was at once passed, while the executive committee was instructed to engage physicians and nurses, and to extend to the stricken father every assistance possible. The speaker in possession of the floor declined to give way for a motion of adjournment, but so shocked were the members at the announcement that the meeting soon adjourned itself, through the members leaving the hall almost in a body. At the time of writing this notice all will be glad to know that, almost against hope, the child is in a fair way to recover, although it has been impossible to remove him from Saratoga.

P. S.—A letter from Dr. Clifton desires us to express his deep sense of gratitude to those members of the Association who evinced such tender interest in his great misfortune, to those who were called upon to render professional service, and especially to Mr. Clair, the proprietor of the Grand Union Hotel, who has been unremitting in his attentions, and unwearied in his kindness. An attempt will be made to remove the boy to his home about the first of September.

